# ASO Author Reflections: Future Directions in Prehabilitation Research

**DOI:** 10.1245/s10434-023-14304-7

**Published:** 2023-09-29

**Authors:** Pratik Raichurkar, Cherry Koh, Daniel Steffens

**Affiliations:** 1 Surgical Outcomes Research Centre (SOuRCe), Sydney Medical School, Royal Prince Alfred Hospital (RPAH), Sydney, NSW Australia; 2 Institute of Academic Surgery (IAS), Royal Prince Alfred Hospital (RPAH), Sydney, Australia; 3 Faculty of Medicine and Health, Central Clinical School, The University of Sydney, Sydney, Australia; 4 Colorectal Department, Royal Prince Alfred Hospital (RPAH), Sydney, Australia

## Past

Surgery is the main curative treatment option for selected patients presenting with cancer. Despite the significant survival benefit, the rates of postoperative morbidity following cancer surgery is high. This results in increased length of hospital stay, decrease in quality of life outcomes, and increase in hospital costs. Prehabilitation aims to provide targeted preoperative interventions, including medical optimization, exercise, nutrition, and psychological support, to improve patient’s health with the intent of reducing postoperative morbidity and enhancing recovery.^1^ Over the last decade, a number of randomized controlled trials and systematic reviews with meta-analysis have established a promising evidence base knowledge, demonstrating significant reductions on postoperative complications and length of hospital stay,^2^ however, this field remains relatively infant. Recent studies have identified heterogenicity in reporting and interventions as barriers to establishing high-quality evidence in this growing field.^3,4^ Therefore, consensus on prehabilitation research priorities could encourage further research in focused areas that are understudied, providing better direction for new investigations.

## Present

The Delphi methodology is a validated tool used to gain consensus on a particular topic from a variety of experts. This study employed a Delphi methodology to establish the top research priorities for prehabilitation in cancer surgery. A total of 165 prehabilitation experts, spanning surgeons, anesthesiologists, physiotherapists, dieticians, psychologists, and specialist nurses, were surveyed. Over the course of two rounds of surveys and voting through an online platform, an initial list of 75 research priorities were aggregated to a final list of 10 key research priorities. The top three research priorities included “Effect of prehabilitation on surgical outcomes,” “Optimal composition of prehabilitation programs,” and “Identifying populations most likely to benefit from prehabilitation.” These priorities reflect modern trends in prehabilitation as well as deficits in current literature.^5^

## Future

This international consensus on the top prehabilitation research priorities is vital, as it allows the research community to focus efforts and resources into areas of most benefit. As the evidence base for prehabilitation grows, it may be applied to patients with cancer undergoing surgery more broadly, better preparing patients for surgery and accelerating their recovery. As this field evolves, we propose that a similar Delphi be performed in 5–10 years to focus research efforts on up-to-date priorities.
